# The MGF300-2R Protein of African Swine Fever Virus Promotes IKKβ Ubiquitination by Recruiting the E3 Ubiquitin Ligase TRIM21

**DOI:** 10.3390/v16060949

**Published:** 2024-06-12

**Authors:** Zhanhao Lu, Rui Luo, Jing Lan, Shengmei Chen, Hua-Ji Qiu, Tao Wang, Yuan Sun

**Affiliations:** 1 State Key Laboratory for Animal Disease Control and Prevention, National African Swine Fever Para-Reference Laboratory, National High Containment Facilities for Animal Diseases Control and Prevention, Harbin Veterinary Research Institute, Chinese Academy of Agricultural Sciences, Harbin 150069, China; 2 College of Animal Sciences, Yangtze University, Jingzhou 434000, China; 3 College of Life Science and Engineering, Foshan University, Foshan 528000, China

**Keywords:** African swine fever virus, MGF300-2R, E3 ubiquitin ligase, TRIM21, ubiquitin, IKK*β*

## Abstract

African swine fever (ASF) is an acute, hemorrhagic, highly contagious disease in pigs caused by African swine fever virus (ASFV). Our previous study identified that the ASFV MGF300-2R protein functions as a virulence factor and found that MGF300-2R degrades IKK*β* via selective autophagy. However, the E3 ubiquitin ligase responsible for IKK*β* ubiquitination during autophagic degradation still remains unknown. In order to solve this problem, we first pulled down 328 proteins interacting with MGF300-2R through immunoprecipitation-mass spectrometry. Next, we analyzed and confirmed the interaction between the E3 ubiquitin ligase TRIM21 and MGF300-2R and demonstrated the catalytic role of TRIM21 in IKK*β* ubiquitination. Finally, we indicated that the degradation of IKK*β* by MGF300-2R was dependent on TRIM21. In summary, our results indicate TRIM21 is the E3 ubiquitin ligase involved in the degradation of IKK*β* by MGF300-2R, thereby augmenting our understanding of the functions of MGF300-2R and offering insights into the rational design of live attenuated vaccines and antiviral strategies against ASF.

## 1. Introduction

African swine fever (ASF) is an acute, hemorrhagic and severe infectious disease of pigs, which has become endemic or epidemic in nearly 50 countries in Africa, Europe, Asia and the Caribbean, causing huge economic losses that are difficult to estimate and posing a critical threat to the global pig industry and related industries [1,2,3]. The causative agent, African swine fever virus (ASFV), is a large, double-stranded DNA virus. The ASFV genome encodes 68 structural proteins and more than 100 non-structural proteins, with over 10 virulence factors discovered in the past five years, such as MGF300-2R, MGF300-4L, A137R, and H240R [2,4,5,6,7]. With the deletion of virulence-associated factors, attenuated strains that can induce protective immune responses against virulent parental virus challenge in pigs are promising live attenuated vaccines (LAVs) [8]. Compared with other types of ASF vaccines, LAVs can provide complete homologous and partial heterologous protection [8]. Hence, it is essential to discover and characterize more virulence-associated genes of ASFV for the future rational development of LAVs.

Selective autophagy degrades specific substrates mediated by selective autophagy receptors (SARs) [9]. The recognition of substrates by ubiquitin-dependent SARs depends on the ubiquitination of substrates by E3 ubiquitin ligases [10]. Accumulating evidence has demonstrated that viruses can regulate selective autophagy by recruiting SARs or E3 ubiquitin ligases to inhibit the host immune responses or promote viral replication. For example, the pseudorabies virus (PRV) tegument protein UL13 recruits the E3 ubiquitin ligase RNF5 to inhibit STING-mediated antiviral immunity [11]. Porcine reproductive and respiratory syndrome virus (PRRSV) degrades DDX10 via SAR p62-dependent selective autophagy to antagonize its antiviral activity [12]. The tegument protein UL21 of alpha-herpesviruses inhibits innate immunity by triggering cGAS degradation through the TOLLIP-mediated selective autophagy [13]. In our previous report, the highly virulent strain ASFV HLJ/18 (ASFV-WT) lacking the *MGF300-2R* gene (Del2R) showed a 1-log reduction in viral titer and induced higher IL-1*β* and TNF-*α* production in primary porcine alveolar macrophages than ASFV-WT. Mechanistically, the MGF300-2R protein interacts with and degrades IKK*α* and IKK*β* via the selective autophagy pathway. Furthermore, we showed that MGF300-2R promoted the K27-linked polyubiquitination of IKK*α* and IKK*β*, which subsequently served as a recognition signal for the selective autophagic degradation mediated by TOLLIP. Importantly, Del2R exhibited a significant reduction in both replication and virulence compared with ASFV-WT in pigs, likely due to increased IL-1*β* and TNF-*α* [6]. However, the E3 ubiquitin ligase responsible for IKK*β* ubiquitination mediated by MGF300-2R remains unknown.

In this study, we conducted immunoprecipitation-mass spectrometry (IP-MS) and confirmed that TRIM21 acts as the E3 ubiquitin ligase responsible for the autophagic degradation of IKK*β* by MGF300-2R, thereby facilitating the understanding of the mechanism underlying the MGF300-2R involvement in the pathogenicity of ASFV. These findings will provide valuable clues to the rational design of LAVs and antiviral strategies against ASF.

## 2. Materials and Methods

### 2.1. Cell Lines

Human embryonic kidney 293T (HEK293T) cells were grown in Dulbecco’s modified Eagle’s medium (DMEM) (C11995500CP; Thermo Fisher Scientific; Waltham, MA, USA) enriched with 10% fetal bovine serum (FBS) (F8687; Sigma-Aldrich; St. Louis, MO, USA) and incubated at 37 °C in a 5% CO_2_ environment. Using the CRISPR/Cas9 system, we generated *TRIM21*-knockout (KO) HEK293T cells by inserting the sgRNA (sg*TRIM21*: 5′-TCTCTCAGGTTGGGAAAGGT-3′) into the plasmid vector pX458.

### 2.2. Antibodies, Plasmids and Reagents

We purchased the following antibodies from Abclonal (Wuhan, China): rabbit anti *β*-tubulin (A12289), mouse anti HA-Tag (AE008), rabbit anti HA-Tag (AE105), mouse anti Flag-Tag (AE005) and mouse anti His-Tag (AE003). Anti-FLAG(R) M2 magnetic beads (M8823) and anti-HA agarose (A2095) were obtained from Sigma-Aldrich (USA). We procured Alexa Fluor 647-conjugated donkey anti-mouse IgG (H+L) (A31571) and Alexa Fluor 488-conjugated goat anti-rabbit IgG (H+L) (A11008) from Thermo Fisher Scientific. 4’, 6-Diamidino-2-phenylindole (DAPI) (C006) was procured from Solarbio (Beijing, China). The TRIM21 polyclonal antibody (12108-1-AP) was obtained from Proteintech (Chicago, IL, USA). The plasmids pHA-MGF300-2R, pFlag-MGF300-2R, pFlag-IKK*β*, and pHis-Ub and the vectors pCMV-HA and pCMV-Flag have been described previously [6]. pHA-IKK*β* was generated by mutating pFlag-IKK*β* using polymerase chain reaction (PCR) site-directed mutagenesis. The pHA-TRIM21 plasmid (ZK3596) was procured from Beijing Zoman Biotechnology and subsequently modified through PCR site-directed mutagenesis to generate pFlag-TRIM21. The primers utilized in this study are summarized in Table 1.

### 2.3. Transfection

HEK293T cells were transfected with indicated plasmids using linear 25-kDa polyethyleneimine (PEI) (23966-2; Polysciences; Warrington, PA, USA). When the cells reached 80% confluence in 6-well cell culture plates, a transfection mixture containing 3 μg of plasmid, 9 μL of PEI (stock solution at 1 mg/mL) and 200 μL of Opti-MEM (31985070; Invitrogen; Carlsbad, CA, USA) was added to the cells and incubated for 24 to 36 h after mixing for 20 min at room temperature.

### 2.4. Immunoprecipitation-Mass Spectrometry (IP-MS) and Proteomics Functional Analysis

In order to analyze the proteins that interact with MGF300-2R, we first verified the MGF300-2R in the immunoprecipitates. Then, the IP-MS service was commissioned by the Beijing Genomics Institute. We conducted a Kyoto Encyclopedia of Genes and Genomes (KEGG) database search and Gene Ontology (GO) enrichment analysis encompassing the cellular component (CC), molecular function (MF) and biological process (BP) categories using clusterProfiler v4.3. The protein–protein interaction (PPI) network was constructed in the STRING database (https://string-db.org/ accessed on 22 December 2023) and visualized via Cytoscape software (version 3.9.1).

### 2.5. Western Blotting and Co-Immunoprecipitation (Co-IP)

HEK293T cells were collected and lysed in ice-cold RIPA lysis buffer (R0278; Sigma-Aldrich; St. Louis, MO, USA), which was supplemented with a protease inhibitor cocktail (4693116001; Roche; Basel, Switzerland). The cell lysates were denatured by boiling them in SDS-PAGE loading buffer (P1040; Solarbio; Beijing, China) and then separated using SDS-PAGE. The separated proteins were transferred onto polyvinylidene fluoride membranes, which were then blocked with 5% skim milk in Tris-buffered saline containing 0.05% Tween 20 (TBST) for 1 h at room temperature. The blocked membranes were subsequently probed using the appropriate primary and secondary antibodies. The signals were visualized using an Odyssey imaging system.

The transfected HEK293T cells were lysed in RIPA lysis buffer for 30 min on ice. After removing the nuclei through low-speed centrifugation and collecting 100 μL of the sample as input, the remaining 800 μL lysates were incubated with the appropriate antibody, after which anti-Flag M2 magnetic beads or anti-HA agarose were added and rotated at 4 °C overnight. After incubation, the beads were collected using a magnetic separator and washed three times with phosphate buffer saline (PBS) (PBS-100; Biocomma; Shenzhen, China). The bound proteins were then subjected to Western blotting using the appropriate antibodies.

### 2.6. Laser Confocal Microscopy

HEK293T cells were seeded onto a glass-bottom cell culture dish (801001; NEST; Wuxi, China) coated with poly-L-lysine. The plasmids were transfected into the cells using PEI. At 24 h post-transfection (hpt), the cells were fixed with 4% paraformaldehyde at room temperature for 20 min, followed by permeabilization with 0.1% Triton X-100 (V900502; Sigma-Aldrich; St. Louis, MO, USA) in PBS for 20 min and blocked with 5% bovine serum albumin (BSA). The primary antibodies were carefully added and allowed to incubate for 2 h. The cells were thoroughly washed with PBS five times, followed by staining with the Alexa Fluor-conjugated secondary antibodies (goat anti-mouse IgG or goat anti-rabbit IgG) for 1 h at room temperature. The cells were washed with PBS three times and then stained with DAPI for 5 min to visualize the nuclei. Following three PBS washing steps, the cells were visualized using confocal laser scanning microscopy with Airyscan (LSM800; Zeiss; Jena, Germany), and the most representative images were chosen for presentation. The scale bar represents 20 μm.

### 2.7. Cell Viability Assay

We utilized a cell counting kit-8 (CCK-8) (CK04; DOJINDO; Kumamoto, Japan) to evaluate the cell viability, following the manufacturer’s guidelines. In summary, the *TRIM21*-knockout (*TRIM21*^−/−^) or wild-type (WT) HEK293T cells were incubated in 96-well cell culture plates. The cell viability was assessed at 12, 24 and 36 h post-incubation (hpi). We added a volume of 10 μL of CCK-8 reagent to each well in the plates. The cells were incubated at 37 °C for 1 h, and subsequently, the absorbance at 450 nm was determined using a microplate reader.

### 2.8. Statistical Analysis

An unpaired two-tailed *t*-test was conducted using GraphPad Prism version 8.0.0 (GraphPad Software, San Diego, CA, USA) to assess statistical significance. The data are presented as the mean ± SD from three independent experiments. *P* ≥ 0.05 was considered statistically non-significant (ns); *P* < 0.05 was considered statistically significant (* *P* < 0.05; ** *P* < 0.01; *** *P* < 0.001).

## 3. Results

### 3.1. The Proteins Interacting with MGF300-2R Were Screened by IP-MS

**Figure 1:**
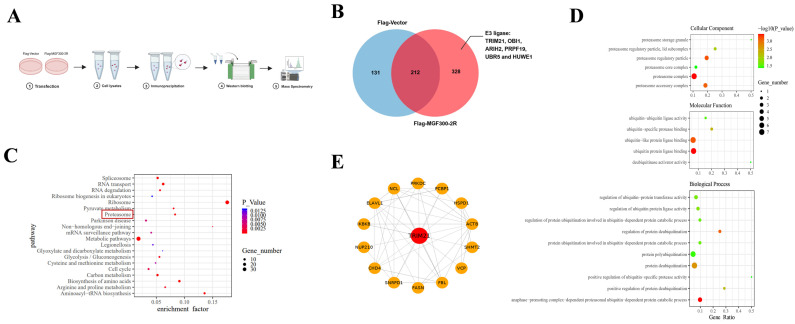
The E3 ubiquitin ligases pulled down by MGF300-2R were screened through Gene Ontology (GO), Kyoto Encyclopedia of Genes and Genomes (KEGG) pathway enrichment and the protein–protein interaction network. (**A**) Obtaining and analyzing the immunoprecipitation samples containing the proteins pulled down by MGF300-2R. (**B**) Venn diagram counted 328 different proteins pulled down in the HEK293T transfected with pFlag-MGF300-2R compared with the HEK293T transfected with vector pCMV-Flag. (**C**,**D**) KEGG pathway analysis and GO category functional enrichment according to three categories (cellular components, molecular functions and biological processes) were performed for 328 proteins. (**E**) The protein–protein interaction network of the proteins that interacted directly with TRIM21 was analyzed against the STRING database.

To identify the E3 ubiquitin ligase responsible for the selective autophagic degradation of IKK*β* by MGF300-2R, we initially transfected HEK293T cells with pFlag-MGF300-2R and subsequently performed immunoprecipitation using magnetic beads coupled with a Flag antibody. The proteins co-immunoprecipitated with MGF300-2R were subjected to IP-MS analysis to identify potential E3 ubiquitin ligases (Figure 1A). Our results indicated a total of 328 proteins pulled down by MGF300-2R, among which the key candidates exhibiting E3 ubiquitin ligase activity included HUWE1, ARIH2, TRIM21, PRPF19, UBR5 and OBI1 (Figure 1B and Table S1). Further Kyoto Encyclopedia of Genes and Genomes (KEGG) pathway analysis indicated the involvement of multiple signaling pathways, such as the proteasome system, the spliceosome system and the RNA transport system (Figure 1C).

In the Gene Ontology (GO) analysis of the IP-MS results, we observed a significant proportion of molecules associated with the ubiquitin–proteasome system (*P* < 0.05) across cellular components (CC), molecular function (MF) and biological processes (BP) (Figure 1D). While HUWE1, UBR5, PRPF19, ARIH2 and OBI1 are mainly related to tumor suppression, DNA damage response and DNA replication [14,15,16,17,18], TRIM21 is involved in regulating host innate immunity and viral immune evasion [19]. Moreover, the protein–protein interaction analysis revealed a direct interaction between TRIM21 and IKK*β* (Figure 1E). Therefore, based on these findings, we selected the E3 ubiquitin ligase TRIM21 for further validation.

### 3.2. The E3 Ubiquitin Ligase TRIM21 Interacts with MGF300-2R

**Figure 2:**
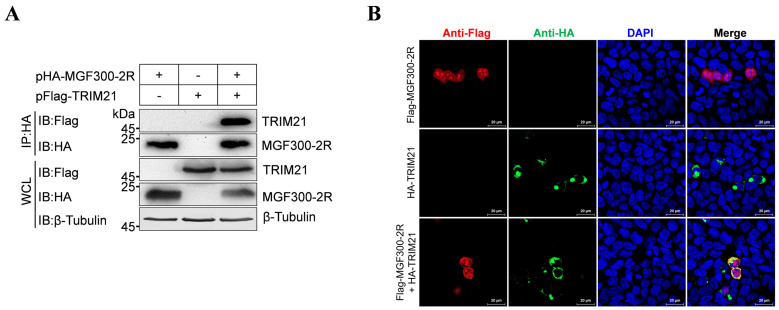
The E3 ubiquitin ligase TRIM21 interacts with MGF300-2R. (**A**) TRIM21 interacts with MGF300-2R. HEK293T cells were transfected with pHA-MGF300-2R and pFlag-TRIM21 alone or co-transfected with them. The whole cell lysates (WCLs) were immunoprecipitated with anti-HA monoclonal antibody at 36 h post-transfection. The immunoprecipitates were examined by Western blotting with the indicated antibodies. (**B**) TRIM21 co-localizes with MGF300-2R. HEK293T cells were transfected with pFlag-MGF300-2R or pHA-TRIM21 alone or co-transfected with pHA-TRIM21 in combination with pFlag-MGF300-2R. TRIM21 and MGF300-2R were analyzed by laser confocal microscopy. Scale bar, 20 μm.

To validate the interaction between MGF300-2R and TRIM21, we performed a co-IP assay. The results showed that TRIM21 was precipitated by MGF300-2R in the lysates from the HEK293T cells co-transfected with pHA-MGF300-2R and pFlag-TRIM21 but not in those of the control group (Figure 2A). Moreover, after co-transfection of the HEK293T cells with pFlag-MGF300-2R and pHA-TRIM21, we observed the co-localization of MGF300-2R and TRIM21 in the cytoplasm of the HEK293T cells using laser confocal microscopy (Figure 2B). These data confirm that the E3 ubiquitin ligase TRIM21 interacts with MGF300-2R.

### 3.3. The E3 Ubiquitin Ligase TRIM21 Promotes the Ubiquitination and Degradation of IKKβ

**Figure 3:**
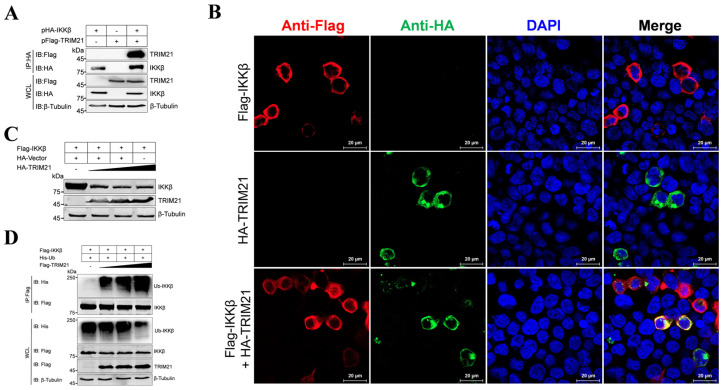
The E3 ubiquitin ligase TRIM21 promotes the ubiquitination and degradation of IKK*β*. (**A**) TRIM21 interacts with IKK*β*. HEK293T cells were transfected with a plasmid encoding HA-IKK*β* along with a plasmid encoding Flag-TRIM21 or vector pCMV-Flag as indicated. The cells were lysed, and the whole cell lysates (WCLs) were immunoprecipitated with anti-HA monoclonal antibody at 36 h post-transfection (hpt). The immunoprecipitates were examined by Western blotting with the indicated antibodies. (**B**) TRIM21 is colocalized with IKK*β.* HEK293T cells were transfected with pFlag-IKK*β* or pHA-TRIM21 alone or co-transfected with pHA-TRIM21 and pFlag-IKK*β.* TRIM21 and IKK*β* were analyzed by laser confocal microscopy. Scale bar, 20 μm. (**C**) TRIM21 promotes the degradation of IKK*β*. HEK293T cells were co-transfected with pFlag-IKK*β* and an increasing amount of pHA-TRIM21 (0, 1.0, 2.0 and 3.0 μg) for 24 h. The cell lysates were analyzed by Western blotting. (**D**) TRIM21 induces the ubiquitination of IKK*β*. HEK293T cells were co-transfected with pFlag-IKK*β* and pHis-Ub and an increasing amount of pHA-TRIM21 (0, 1.0, 2.0 and 3.0 μg). At 24 hpt, the cells were processed for immunoprecipitations with anti-Flag magnetic beads and analyzed by Western blotting with the indicated antibodies.

To investigate the involvement of the E3 ubiquitin ligase TRIM21 in IKK*β* degradation induced by MGF300-2R, we transfected HEK293T cells with pHA-IKK*β* and pFlag-TRIM21 alone or co-transfected with them. As shown in Figure 3A, TRIM21 immunoprecipitated with IKK*β*. Laser confocal microscopy analysis confirmed that TRIM21 and IKK*β* were colocalized in the cytoplasm of HEK293T cells (Figure 3B), suggesting an interaction between IKK*β* and TRIM21. To ascertain whether TRIM21 has a role in IKK*β* degradation, we co-transfected the HEK293T cells with pFlag-IKK*β* and varying amounts of pHA-TRIM21. The results showed a dose-dependent reduction in IKK*β* protein levels (Figure 3C), indicating TRIM21 facilitates IKK*β* degradation. Moreover, by overexpressing pFlag-IKK*β*, along with pHis-Ub and increasing the doses of pFlag-TRIM21 in the HEK293T cells, followed by co-IP assay, we found the dose-dependent ubiquitination of IKK*β* by TRIM21 (Figure 3D). These results indicate that TRIM21 functions as an E3 ubiquitin ligase, promoting the ubiquitination and subsequent degradation of IKK*β*.

### 3.4. Degradation of IKKβ by MGF300-2R Depends on TRIM21

**Figure 4:**
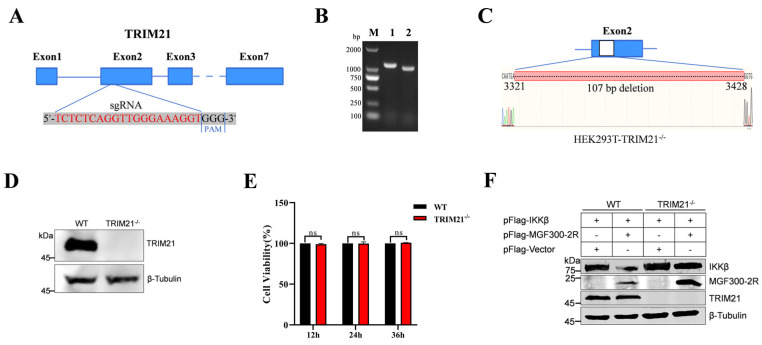
Degradation of IKK*β* by MGF300-2R depends on TRIM21. (**A**) Schematic of the TRIM21-knockout strategy. The sgRNA target sites are indicated in red. (**B**–**D**) HEK293T cells with *TRIM21* knockout (HEK293T-*TRIM21*^−/−^). Confirmation of *TRIM21* knockout in HEK293T cells using PCR, Sanger sequencing and Western blotting. The wild-type HEK239T (HEK293T-WT) cells’ and HEK293T-*TRIM21*^−/−^ cells’ genomes were extracted and subjected to PCR with primers TRIM21-F/R. Lane M displays a DL2,000 DNA marker, and Lanes 1 and 2 show the PCR results of the HEK293T-WT and HEK293T-*TRIM21*^−/−^ cells’ genomes (**B**). The changes in ORF of the *TRIM21* gene were verified by Sanger sequencing by Rui Biotech (**C**). The expression of TRIM21 protein in HEK293T-WT and HEK293T-*TRIM21*^−/−^ cells was verified with anti-TRIM21 polyclonal antibody by Western blotting (**D**). (**E**) HEK293T-*TRIM21*^−/−^ cells are not significantly affected in terms of cell viability compared with HEK293T-WT at different time points (12, 24, and 36 h post-incubation) using CCK-8 detection kit. (**F**) MGF300-2R does not promote the degradation of IKK*β* in HEK239T-*TRIM21*^−/−^ cells. HEK293T-WT and HEK293T-*TRIM21*^−/−^ cells were co-transfected with pFlag-MGF300-2R or vector pCMV-Flag in combination with pFlag-IKK*β*.

To study the role of TRIM21 in the degradation of IKK*β* by MGF300-2R, we generated *TRIM21*-knockout (KO) HEK293T (HEK239T-*TRIM21*^−/−^) cells. Briefly, the targeting RNA (sgRNA) targeting exon 2 of the *TRIM21* gene was designed using the CRISPR Design website (http://crispr.mit.edu, accessed on 24 February 2023) (Figure 4A). The knockout of *TRIM21* was determined by PCR, Sanger sequencing and Western blotting. In comparison with the wild-type HEK239T (HEK293T-WT) cells, the HEK239T-*TRIM21*^−/−^ cells exhibited a deletion of 107 base pairs in the exon 2 sequence of the *TRIM21* gene (Figure 4B,C) and notably lacked TRIM21 protein expression (Figure 4D). Cell viability analysis revealed that knocking out *TRIM21* in the HEK239T cells had no significant effect on the cell viability at 12, 24 and 36 h post-incubation (Figure 4E). Subsequently, to investigate the influence of *TRIM21* deletion in the HEK239T cells on the degradation of IKK*β* by MGF300-2R, both HEK293T-WT and HEK239T-*TRIM21*^−/−^ cells were transfected either with pFlag-IKK*β* alone or co-transfected with pFlag-MGF300-2R and pFlag-IKK*β*. The results demonstrated that MGF300-2R promoted the degradation of IKK*β* in the HEK293T-WT cells, whereas no such phenomenon was observed in the HEK239T-*TRIM21*^−/−^ cells (Figure 4F). These findings conclusively establish TRIM21 as an E3 ubiquitin ligase crucially involved in MGF300-2R-mediated IKK*β* degradation.

## 4. Discussion

**Figure 5:**
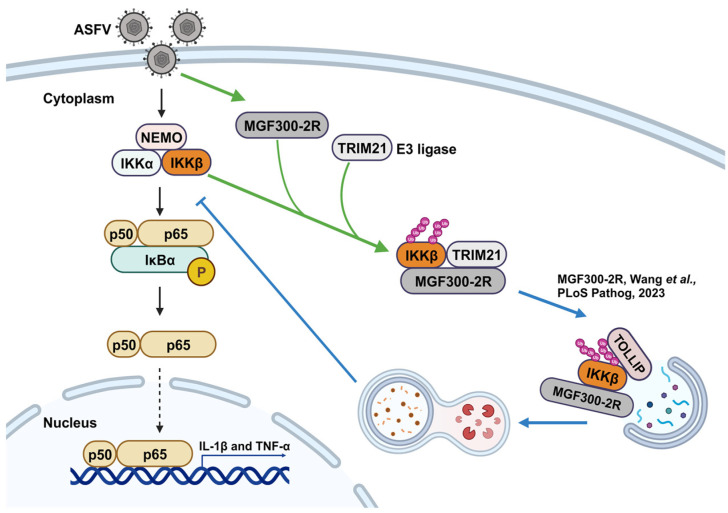
A working model for the recruitment of TRIM21 to promote the ubiquitination and degradation of IKK*β* by the ASFV MGF300-2R protein. The ASFV MGF300-2R protein recruits the E3 ubiquitin ligase TRIM21 to ubiquitinate IKK*β*, and IKK*β* is subsequently recognized and degraded via the selective autophagic pathway, which subverts the NF-*κ*B signaling pathway [6].

ASF LAVs can provide complete homologous and partial heterologous protection [8]. Identifying new virulence factors and analyzing the pathogenic mechanisms involved in virulence factors will contribute to the development of LAVs [20]. Our previous study identified a new virulence factor MGF300-2R and found that it degrades IKK*β* through selective autophagy. Here, we discovered that the E3 ubiquitin ligase TRIM21 is involved in the degradation of IKK*β* by MGF300-2R. Through the IP-MS results, we pulled down 328 proteins that can interact with MGF300-2R (Figure 1). The screened E3 ubiquitin ligase TRIM21 can interact with MGF300-2R and IKK*β* and promote IKK*β* ubiquitination and degradation (Figure 2 and Figure 3). After the knockout of *TRIM21*, we demonstrated that the degradation of IKK*β* by MGF300-2R is dependent on TRIM21 (Figure 4). Therefore, we conclude that ASFV MGF300-2R promotes IKK*β* autophagic degradation by recruiting the E3 ubiquitin ligase TRIM21 (Figure 5).

The E3 ubiquitin ligase can promote the ubiquitination and degradation of viral proteins to resist viral infection [19,21]. On the contrary, the viruses antagonize antiviral responses by recruiting the E3 ubiquitin enzyme [11]. Our results show that ASFV MGF300-2R recruits TRIM21 to antagonize NF-*κ*B. Additionally, studies have reported that ASFV has at least seven proteins that directly recruit E3 ubiquitin ligases in innate immunity to promote the degradation of regulatory factors by ubiquitination, indicating that the recruitment of E3 ubiquitin enzymes may be one important strategy for ASFV to antagonize the host immune defense [22,23,24,25,26,27,28]. TRIM21 plays essential roles in substrate binding and ubiquitination by two conservative domains, RING and PRY-SPRY [29]. The human papillomavirus (HPV) oncoprotein E7 can enhance the interaction between the PRY/SPRY domain of TRIM21 and the PYD domain of IFN-*γ* inducible protein 16 (IFI16), catalyzing the K33-linked ubiquitination degradation of IFI16, leading to cellular pyroptosis and viral immune escape [30]. Hence, identifying the key functional domains of TRIM21 involved in promoting the MGF300-2R-mediated degradation of IKK*β* warrants further investigation. Studies have shown that the *TRIM21* gene is a host restriction factor for PRRSV and porcine epidemic diarrhea virus (PEDV) infection in porcine alveolar macrophages and foot-and-mouth disease virus (FMDV) in porcine kidney 15 cells [31,32]. Given the important roles of TRIM21 in antiviral response, it is unsurprising that viruses have developed various strategies to counteract its activity.

Overall, our data indicate that TRIM21 functions as the E3 ubiquitin ligase responsible for degrading IKK*β* through ASFV MGF300-2R. This finding highlights the negative regulatory role of TRIM21 recruited by MGF300-2R in the NF-*κ*B signaling pathway. Furthermore, our study enriches the understanding of the mechanism underlying the MGF300-2R involvement in the pathogenicity of ASFV and identifies potential targets for the design of ASF vaccines and the development of antivirals.

## Figures and Tables

**Table 1:** The primers used in this study.

Primers	Sequence (5′-3′)
Flag-TRIM21-Forward	CAAGCTTGCGGCCGCGAATTCGATGGCTTCAGCAGCACGC
Flag-TRIM21-Reverse	ATCAGATCTATCGATGAATTCTCAATAGTCAGTGGATCCTTGTGATC
sgRNA-Forward	TCTCTCAGGTTGGGAAAGGT
sgRNA-Reverse	ACCTTTCCCAACCTGAGAGA
TRIM21-Forward	GCTTTGGCTATTCTGAGTCT
TRIM21-Reverse	TCCCCTTTGAACCTGCATC

## Data Availability

The original contributions presented in the study are included in the article and Supplementary Materials, and further inquiries can be directed to the corresponding authors.

## Supplementary Materials

The following supporting information can be downloaded at https://www.mdpi.com/article/10.3390/v16060949/s1. Table S1: Identification of the proteins interacting with MGF300-2R.
